# Dysregulation of MicroRNAs in Hypertrophy and Ossification of Ligamentum Flavum: New Advances, Challenges, and Potential Directions

**DOI:** 10.3389/fgene.2021.641575

**Published:** 2021-04-12

**Authors:** Baoliang Zhang, Guanghui Chen, Xiaoxi Yang, Tianqi Fan, Xi Chen, Zhongqiang Chen

**Affiliations:** Orthopaedic Department, Peking University Third Hospital, Beijing, China

**Keywords:** ossification of ligamentum flavum, hypertrophy of ligamentum flavum, microRNAs, pathogenesis, diagnosis, therapy

## Abstract

Pathological changes in the ligamentum flavum (LF) can be defined as a process of chronic progressive aberrations in the nature and structure of ligamentous tissues characterized by increased thickness, reduced elasticity, local calcification, or aggravated ossification, which may cause severe myelopathy, radiculopathy, or both. Hypertrophy of ligamentum flavum (HLF) and ossification of ligamentum flavum (OLF) are clinically common entities. Though accumulated evidence has indicated both genetic and environmental factors could contribute to the initiation and progression of HLF/OLF, the definite pathogenesis remains fully unclear. MicroRNAs (miRNAs), one of the important epigenetic modifications, are short single-stranded RNA molecules that regulate protein-coding gene expression at posttranscriptional level, which can disclose the mechanism underlying diseases, identify valuable biomarkers, and explore potential therapeutic targets. Considering that miRNAs play a central role in regulating gene expression, we summarized current studies from the point of view of miRNA-related molecular regulation networks in HLF/OLF. Exploratory studies revealed a variety of miRNA expression profiles and identified a battery of upregulated and downregulated miRNAs in OLF/HLF patients through microarray datasets or transcriptome sequencing. Experimental studies validated the roles of specific miRNAs (e.g., miR-132-3p, miR-199b-5p in OLF, miR-155, and miR-21 in HLF) in regulating fibrosis or osteogenesis differentiation of LF cells and related target genes or molecular signaling pathways. Finally, we discussed the perspectives and challenges of miRNA-based molecular mechanism, diagnostic biomarkers, and therapeutic targets of HLF/OLF.

## Introduction

Pathological changes in the ligamentum flavum (LF) can be defined as a process of chronic progressive aberrations in the nature and structure of ligamentous tissues, characterized by increased thickness, reduced elasticity, local calcification, or aggravated ossification of LF fibroblasts, which may cause spinal stenosis and severe myelopathy, radiculopathy, or both (Yayama et al., 2007; Yabe et al., 2015; Sugimoto et al., 2018). Clinically, hypertrophy of ligamentum flavum (HLF) and ossification of ligamentum flavum (OLF) are the main pathological categories, while calcification of ligamentum flavum (CLF) is extremely rare (Giulioni et al., 2007). Histopathologically, it has been proposed that HLF and OLF, in its essence, are the process of fibrosis and endochondral osteogenesis of ligamentum fibroblasts under numerous external stimuli (Yayama et al., 2007; Sun et al., 2020). Extensive evidence has shown that genetic background (Hou et al., 2014; Zhang C. et al., 2017), mechanical stress (Hayashi et al., 2017; Shunzhi et al., 2017), aging and gender (Safak et al., 2010; Moon et al., 2015; Kim et al., 2018), endocrine and metabolic abnormalities (Dario et al., 2015; Shemesh et al., 2018; Chaput et al., 2019), local inflammation, and angiogenesis (Zhang K. et al., 2017; Sun et al., 2018; Yang et al., 2018a; Jezek et al., 2020) are potential predisposing factors in the development of HLF/OLF. In addition, intrinsic alterations in multiple cellular activities, growth factors, and molecular mediators have been implicated in this intricate process (Chao et al., 2016; Qu et al., 2016a; Sidon et al., 2019; Ye et al., 2019). However, the definitive pathogenesis remains largely unclear.

During the last decade, epigenetic regulations have been considered as a significant molecular mechanism that can modulate genome activity and cause phenotype changes without any alterations of the underlying genotype (Skinner et al., 2010; Brookes and Shi, 2014), which can link genetic and environmental risk factors for diseases, uncover gene–environment interactions, discover valuable biomarkers, and explore potential therapeutic targets (Ladd-Acosta and Fallin, 2016; Berdasco and Esteller, 2019; Cavalli and Heard, 2019). MicroRNAs (miRNAs), the most widely investigated epigenetic modifications, have been demonstrated to be involved in the pathogenesis of ligamentum flavum tissue hypertrophy and ossification. MiRNAs are evolutionarily conserved single-stranded, non-coding RNA molecules comprising 19–25 nucleotides (Jung and Suh, 2014). To date, more than 2,000 functional miRNAs have been determined, and the expression of about 60% of the human gene is regulated by these miRNAs at the posttranscriptional level (Kloosterman and Plasterk, 2006; Silahtaroglu and Stenvang, 2010). Functionally, miRNAs can constitute the RNA-induced silencing complex and inhibit translation or induce degradation of mRNA through base-pairing rules between the complementary sequences of miRNA and its target mRNAs (Gulyaeva and Kushlinskiy, 2016). It has been identified that dysregulated miRNA level is bound up with numerous physiological and pathological processes, including bone homeostasis (bone formation, resorption, remodeling, etc.) (Pi et al., 2015). Afterward, substantial evidence has revealed that miRNAs functioned in various bone and cartilage-related diseases such as fracture (Waki et al., 2015), osteoporosis (Gu et al., 2019), osteoarthritis (Coutinho de et al., 2019), intervertebral disc degeneration (Ji et al., 2018), ossification of posterior longitudinal ligament, (Xu et al., 2019) and osteosarcoma (Andersen et al., 2018), which provided a new direction for researchers to investigate pathogenesis, detect diagnostic biomarkers, and invent treatment modalities (Makeyev and Maniatis, 2008).

**Figure 1:**
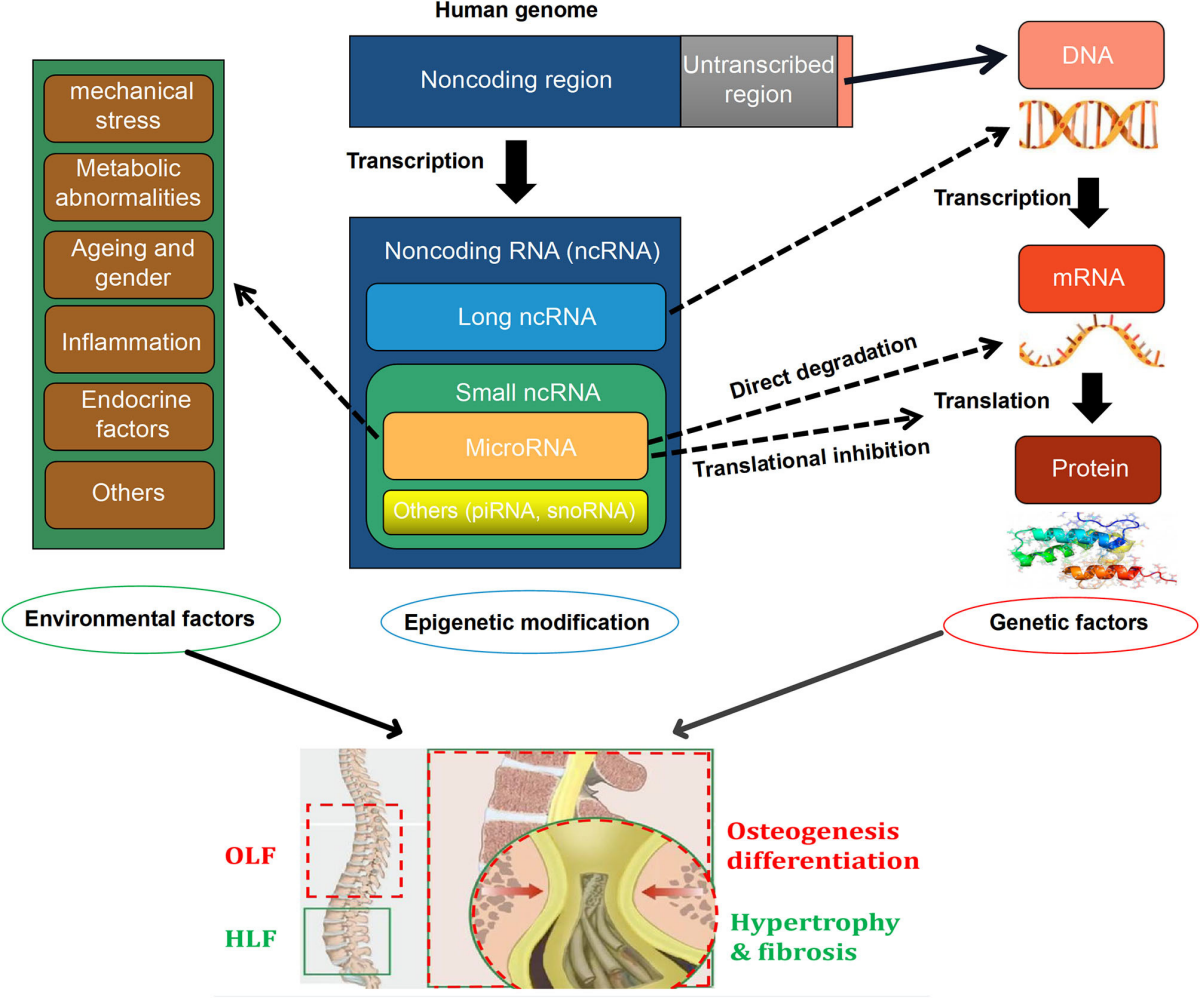
A hypothetical map illustrating that the interactions among epigenetic modifications, [especially microRNAs (miRNAs)], genetics, and environmental factors in the pathogenesis of ossification of ligamentum flavum (OLF) and hypertrophy of ligamentum flavum (HLF).

Currently, emerging efforts have been concentrated on exploring the critical role of miRNAs on the intrinsic mechanism of HLF and OLF, and preliminary results revealed that miRNAs might link genetic and environmental factors with an altered risk of OLF/HLF by targeting specific genes or influencing transcription factors and related molecular signaling. Based on current evidence, a hypothetical map was depicted to briefly elaborate the potential interrelation among epigenetic regulations, genetic background, and environmental factors, and the biological importance of miRNAs in the pathomechanism of OLF/HLF (Figure 1). Besides involvement of mechanistic studies, miRNAs also possess potential applications as diagnostic biomarkers because specific miRNAs seem to have a disturbed expression in several cells and body compartments in patients with bone-related disorders (Bottani et al., 2020). Furthermore, miRNAs have a distinct advantage as novel therapeutic targets with the possibility of avoiding undesirable side effects, which may provide a novel sight into tailored therapies for HLF/OLF (Gao et al., 2020).

In terms of the key role of miRNAs in gene expression regulation, it is worthwhile to explore indepthly the underlying functions of miRNA and its feasible regulating networks in occurrence and development of HLF and OLF. Therefore, this study aimed to provide a comprehensive overview of current publications concerning the expression profiling and functional characterization of miRNAs associated with ligamentum flavum hypertrophy and ossification, and highlight those significant dysreguled miRNAs and their target genes/related signaling pathways in pathological processes of LF cells, and emphatically discuss perspectives and challenges of miRNAs as potential biomarkers or novel therapeutic targets for HLF/OLF in further researches.

## Expression Profiling of Micrornas in Ossification of Ligamentum Flavum

Han et al. (2018) investigated the miRNA expression profiles in OLF samples compared with non-OLF samples through miRNA sequencing and identified 28 altered miRNAs (fold change >2, *p* < 0.05). Furthermore, five upregulated miRNAs (miR-181a-5p, miR-181a-3p, miR-707-5p, miR-181b-5p, and miR-146a-5p) and five downregulated miRNAs (miR-889-3p, miR-32-5p, miR-379-5p, miR-381-3p, and miR-19b-3p) were confirmed by qRT-PCR. Subsequently, miR-19b-3p, the significantly expressed miRNA, was markedly decreased in OLF cells and in human mesenchymal stem cells (hMSCs) under osteogenic induction, and overexpressed miR-19b-3p could inhibit the levels of the osteogenic differentiation-related genes (RUNX2, COL1a1, and ALP), which indicated that miR-19b-3p was involved in the development of OLF. Additionally, a miRNA-19b-3p-based miRNA–circRNA–lncRNA–mRNA network (lncRNA ENST00000608133 and ENST00000599584, miR-19b-3p, and circRNA circ_0050139) was preliminarily established in the process of ossification for the first time, which provided an insight into the interplay of miRNAs and other noncoding RNAs in osteogenic differentiation of LF cells.

Afterward, Kong et al. (2019) used the public datasets to further identify 81 differentially expressed miRNAs (DEMs) between four TOLF samples and four normal controls and screened crucial miRNAs in the pathogenesis of OLF based on the miRNA–mRNA and lncRNA–miRNA–mRNA competing endogenous RNA (ceRNA) regulatory networks. On one hand, the miRNA–mRNA network analysis demonstrated that downregulated miR-379-5p in OLF was significantly connected with increased proinflammatory marker GNG4, while upregulated miR-210-3p, miR-196a-5p, and miR-181b-5p were significantly associated with decreased target IL10, SOCS3, and ADCY5, respectively, all of which are anti-inflammatory markers. On the other hand, the ceRNA network analysis identified that miR-329-3p and miR-222-5p regulated the osteogenic differentiation of LF cells by targeting COL13A1 and COL2A1, respectively. In addition, miR-299-3p, which was competitively combined with RHPN1-AS1, targeted WNT7B and modulated the Wnt signaling pathway during ossification. Similarly, in the study by Yayama et al. (Yayama et al., 2018), three DEMs met the given criteria (*p* < 0.05 and log2 ratio >1) among 12 downregulated miRNAs, namely, miR-137, miR-382-5p, and miR-487b-3p. Furthermore, miR-487b-3p was predicted to play a vital role in the activation of Wnt signaling during the LF ossification process.

Wu et al. (Wu et al., 2020) further conducted a comprehensive bioinformatic analysis on three datasets (GSE106253, GSE106256, and GSE106255) deposited by Hong et al. to distinguish DEMs associated with TOLF. Fifteen upregulated miRNAs and 14 downregulated miRNAs were determined to be significantly differentially expressed in OLF tissues compared with normal controls. COL6A1 from the 17 OLF-related genes (NPPS, COL11A2, BMP2, BMP4, BMP9, TGF-b1, etc.) was the overlapping gene in the constructed lncRNA- and circRNA-related ceRNA network, which might influence the development of OLF.

## Expression Profiling of MICRORNAs in Hypertrophy of Ligamentum Flavum

Microarray-based profiling of miRNAs in HLF was first performed by Xu and colleagues, and 538 miRNAs were screened preliminarily by microRNA array (Xu Y. Q. et al., 2016). Furthermore, 18 DEMs (15 upregulated and three downregulated) were identified in the hypertrophied LF compared with the normal control LF. When miRNAs with a mean fold change more than 2 or <0.5 and a *P* < 0.01 were selected for further analysis, miR-202-3p (2.6-fold, *P* = 0.3), miR-486 (3.8-fold, *P* = 0.06), and miR-221 (0.2-fold, *P* = 0.008) were determined to be significantly dysregulated. For further verification, researchers detected the level of these three candidate miRNAs in a larger samples involving 38 patients and 22 controls by qRT-PCR. Eventually, the expression of miR-221 was significantly lower in patients with LSS compared with the controls, which was consistent with those in the training set.

Based on transcriptome sequencing, deregulated miRNAs profiling in HLF patients was conducted by Mori et al. in which they utilized the Agilent microarray and identified 10 DEMs in hypertrophied LF tissues (*n* = 10) vs. normal tissues (*n* = 10) (Mori et al., 2017). In this expression signature, nine miRNAs (miR-1228-3p, miR-1237, miR-30c-2-3p, miR-423-5p, miR-4306, miR-483-5p, miR-514b-5p, miR-516b-5p, and miR-765) were downregulated, and only one (miR-497-5p) was upregulated in the hypertrophied ligaments. In addition, data analysis found that the levels of miR-29c-3p, miR-595, miR-663b, miR-1290, and miR-223-3p were significantly related to donor age, while miR-423-5p, miR-4306, miR-516b-5p, and miR-497-5p were associated with the ratio of LF/spinal canal area based on MRI T2 measurements. Further pathway analysis revealed that Wnt/β-catenin signaling, aryl hydrocarbon receptor signaling, and insulin receptor signaling were highly implicated in the fibrosis and hypertrophy of LF cells predicted by miRNA signature.

## Functional Characterization of Specific MICRORNAs in Ossification of Ligamentum Flavum

### MiR-132-3p

Qu et al. (2016b) demonstrated that the level of miR-132-3p was decreased in OLF samples compared with healthy controls. Osteogenic differentiation was significantly suppressed by overexpression of miR-132-3p, while transfection of inhibitors of miR-132-3p significantly promoted osteogenic activity of LF cells. FOXO1, GDF5, SOX6, three osteogenesis-related genes, were identified as the direct targets of miR-132-3p. Upregulated miR-132-3p induced the reduced expression of FOXO1, GDF5, and SOX6, but the downregulation of miR-132-3p generated opposite effects. Meanwhile, the level of these three genes was increased under osteogenic induction, and their downregulation restrained the osteogenic differentiation of LF cells. These evidence collectively suggested that miR-132-3p could mediate the ossification process of the ligamentum flavum by targeting FOXO1, GDF5, and SOX6, thus miR-132-3p could possibly be considered as available therapeutic targets for OLF. Admittedly, the miR-212/132 family belongs to highly conserved noncoding RNAs in vertebrates (Bicker et al., 2014). Consistently, an integrated study also identified miR-132-3p as one of the top 10 downregulated miRNAs in OPLL samples compared with those in the controls (Xu C. et al., 2016). In addition, previous studies suggested that miR-132-3p could inhibit osteoblast differentiation under simulated microgravity environment and in type 2 diabetes mellitus-induced osteoporosis (Hu et al., 2015; Gong et al., 2016). Recently, miR-132-3p was found to be influenced by lncRNA APTR and LncRNA TUG1 in the regulation of osteosarcoma, a tumor that has an osteogenic capability (Li G. et al., 2018; Guan et al., 2019). These findings will provide new references into improving the regulation mechanism of miR-132-3p in OLF.

### MiR-199b-5p

Qu et al. (2017) further observed that miR-199b-5p was another dramatically downregulated miRNA during OLF, and upregulated miR-199b-5p could hold back the ossification process. Moreover, JAG1 was identified as a direct target of miR-199b-5p through dual-luciferase reporter assays. Meanwhile, JAG1 is a crucial Notch ligand and functions in the Notch signaling pathway. Furthermore, miR-199b-5p could inhibit the expression level of JAG1 and Notch, whereas JAG1 knockdown blocked the inhibitory effect of miR-199b- 5p. These results concluded that miR-199b-5p exerted an inhibitory effect on osteogenic differentiation of ligamentum fibroblasts by potentially targeting JAG1 and via the Notch signaling pathway. Concordantly, miR-199b-5p has been also recognized as one of obviously downregulated miRNAs in OPLL tissues, and was predicted to regulate JAG1 (Xu C. et al., 2016). Contrarily, another study found that the reduced expression of miR-199b-5p was examined in the osteogenic differentiation of bone marrow stromal cells (BMSCs) via suppression of the GSK-3β/β-catenin signaling pathway (Zhao et al., 2016). It was speculated that miR-199b-5p might perform a bidirectional regulatory function on osteogenic differentiation of various cell types.

### MiR-615-3p

A study by Yin et al. (2017) showed that miR-615-3p was downregulated during the osteogenic differentiation of LF cells. Then, gain- and loss-function experiments demonstrated that miR-615-3p negatively regulated the ossification process with a lighter Alizarin Red staining and a decreased expression of ALP, RUNX2, Ostx, OCN, and OPN. Subsequently, FOXO1 and GDF5 were identified as direct target of miR-615-3p by luciferase activity assay and bioinformatic analysis, and miR-615-3p could inhibit the expression of FOXO1 and GDF5. On the side, knockdown of either FOXO1 or GDF5 could inhibit the osteogenic differentiation. In conclusion, miR-615-3p negatively modulated the development of ligamentum flavum ossifications through posttranscriptionally targeting GDF5 and FOXO1. It could be recommended as a potential target for human OLF therapy. Moreover, this is the first report to confirm miR-615-3p as a negative regulator in the osteogenic differentiation of various human cell lineages including BMSCs, osteoblasts, and ligamentum fibroblasts, indicating that miR-615-3p might be one of the important human osteogenesis-related miRNAs.

### MiR-490-3p

Yang et al. (2018b) first investigated the function of miR-490-3p in TOLF. MiR-490-3p presented downregulated expression during OLF process, and its overexpression further depressed osteogenic differentiation of ligament fibroblasts. In addition, the fact that miR-490-3p directly targeted FOXO1 was supported by dual-luciferase assays, and miR-490-3p negatively regulated the level of FOXO1. Furthermore, FOXO1 knockdown attenuated the inhibitory effect of miR-490-3p. ChIP assays demonstrated that the interaction of FOXO1 and RUNX2 was inhibited by miR-490-3p. Taken together, upregulated miR-490-3p could be suppressed in osteogenic differentiation of LF cells by potentially targeting FOXO1, indicating that restoring miR-490-3p and restraining FOXO1 might be a potential therapeutic strategy for TOLF. A previous study on miRNA–mRNA suggested that the expression of miR-490-3p was downregulated in OPLL cells, but its exact mechanism was not elucidated (Xu C. et al., 2016). Importantly, this study took the lead to validate the functional role of miR-490-3p in the progression of OLF, which provided important insights into other skeletal diseases.

### MiR-182

Zhang et al. (2018) found that miR-182 was downregulated in OLF tissue compared with non-OLF tissues. Nicotinamide phosphoribosyl transferase (NAMPT) was progressively elevated during osteogenic differentiation of bone marrow-derived mesenchymal stem cells, which might be considered as the osteogenesis marker. Besides, miR-182 overexpression inhibited the expression level of NAMPT, RUNX2, OCN, and OPN in OLF cells. Meanwhile, knockdown of NAMPT and the use of NAMPT inhibitor could downregulate the expression of RUNX2, OCN, and OPN, whereas upregulation of NAMPT led to the opposite effect. Dual-luciferase reporter assays predicted that NAMPT was the direct target of miR-182. Further experiments showed that upregulated miR-182 inhibited the effects of NAMPT overexpression on promoting the mRNA and protein level of RUNX2, OCN, and OPN. Overall, these data demonstrated that miR-182 suppressed OLF by targeting NAMPT. Analogously, Kim et al. revealed that miR-182 could inhibit the proliferation and differentiation of osteoblasts by suppressing FoxO1 (Kim et al., 2012). Moreover, Wang reported that lncRNA POIR and miR-182 formed a competing endogenous RNA (ceRNA) and promoted osteogenic differentiation of periodontal mesenchymal stem cells in periodontitis patients through suppressing FoxO1 (Wang et al., 2016). Furthermore, Kazuki et al. found the regulatory mechanism of the miR-182–PKR–IFN-β axis during osteoclastogenesis process both in *in vitro* and *in vivo* systems, which provided the translational implications of miR-182 as therapeutic target to prevent bone loss (Inoue et al., 2018).

### MiR-29a-5p

Feng et al. (2020) interpreted the function and mechanism of miR-29a-5p and special AT-rich sequence-binding protein 2 (SATB2) in the development of TOLF. In the first place, downregulated miR-29a-5p and upregulated SATB2 were significantly observed in TOLF tissues. Concordantly, miR-29a-5p expression was also decreased during osteogenic differentiation of LF cells, and a prominent reduction in the expression level of key osteogenesis markers was identified with the overexpression of miR-29a-5p. On the contrary, this process was enhanced when miR-29a-5p was inhibited. Furthermore, the phenomenon that miR-29a-5p directly targeted SATB2 and suppressed its expression was validated. Knockdown of SATB2 distinctly impeded the effects of miR-29a-5p on inhibiting osteogenesis, and this also contributed to SIRT1 downregulation and Smad3 acetylation. To sum up, these findings indicated that miR-29a-5p could effectively inhibit thoracic LF cell osteogenesis differentiation via targeting SATB2 and through influencing SIRT1/Smad3 deacetylation pathway. Coincidently, miR-29a-5p has been shown to be downregulated in the development of OPLL, which implied that miR-29a-5p might be critical in the pathogenesis of heterotopic ossifying diseases (Xu C. et al., 2016).

## Functional Characterization of Specific MICRORNAs in Hypertrophy of Ligamentum Flavum

### MiR-155

Chen et al. (2014) found that the expression of miR-155 was significantly elevated in hypertrophic LF tissues from LSS groups than in control groups. Moreover, miR-155 level was positively correlated with LF thickness and the level of type I and type III collagen. To test the effects of miR-155 in the regulation of types I and III collagen expression in LF, infection of miR-155 mimic lentivirus contributed to the increased expression of collagen I and collagen III in LF cells, whereas miR-155 sponge lentivirus produced the opposite effect, which implied that miR-155 was a fibrosis-associated miRNA and might play a crucial part in the onset and progression of LF hypertrophy. Emerging evidence has demonstrated that miR-155 functioned in the pathogenesis of various fibrotic diseases (Jiang et al., 2010; Artlett et al., 2017). Additionally, the development of LF hypertrophy was proven to be accompanied by substantial macrophage infiltration (Saito et al., 2017). Interestingly, miR-155 has been recognizable as an important element of the primary macrophage response to inflammatory mediators, which suggested that miR-155 might be involved in immunomodulatory effects during LF hypertrophy (Wang et al., 2011). Furthermore, miR-155 has been verified as a direct target for the TGF-β/Smad pathway, while many studies have shown that TGF-β was implicated in the hypertrophic process of LF, and these findings might demonstrate a potential association among miR-155, TGF-β pathway, and LF hypertrophy (Kong et al., 2008; Löhr et al., 2011).

### MiR-221

As mentioned above, (Xu Y. Q. et al., 2016) performed comprehensive miRNA microarray and identified miR-221 as one of the significantly downregulated miRNAs in degenerative LF tissues. Functionally, overexpression of miR-221 suppressed expression of collagens I and III in LF cells compared with untreated cells. Bioinformatics target prediction revealed that tissue inhibitors of matrix metalloproteinase (TIMP)-2 acted as a putative target of miR-221. Consistently, luciferase reporter assays also demonstrated that miR-221 directly targeted TIMP-2 and reduced the protein expression of TIMP-2 in LF cells. To sum up, downregulated miR-221 might promote LF hypertrophy through inducing collagens I and III expression via targeting TIMP-2. A growing body of evidence showed that miR-221 was one of fibrosis-associated miRNAs and played important parts in the occurrence and development of fibrotic diseases, such as liver fibrosis (Tsay et al., 2019), cardiac fibrosis (Zhou et al., 2020), and renal fibrosis (Morinaga et al., 2016). Therefore, future researches are expected to conduct a further exploration of miR-221 as the potential therapeutic target for HLF.

### MiR-21

Sun et al. (2017) conducted a scientific research to detect upregulated miR-21 in hypertrophic LF tissues compared with normal tissues through RT-PCR analysis. Besides, the dysregulation of miR-21 established positive relations with the LF thickness and fibrosis scores, which meant that the expression level of miR-21 elevated continually along with progressive fibrosis and accumulating thickness. Meanwhile, miR-21 overexpression promoted the levels of collagen I and III in LF cells, thus considering that deregulated miR-21 played a role in the fibrosis of LF. Previous findings (Nakamura et al., 2015; Sun et al., 2018) have shown that IL-6 was significantly increased and could increase collagen expression in LF tissues. Furthermore, Sun and his partners investigated and found that the mRNA and protein expression of IL-6 was upregulated by miR-21 mimic in LF hypertrophy. Taken together, miR-21 was determined as a fibrosis-associated miRNA, which could induce inflammation by activating IL-6 expression in LF tissue, resulting in LF fibrosis and hypertrophy. Like miR-221, accumulated studies have considered microRNA-21 as a central regulator of fibrotic diseases (e.g., hepatic fibrosis, skin fibrosis, and cardiac fibrosis) via various targets or molecular mechanism (e.g., inflammation, immunoreaction, and autophagy) (Yan et al., 2020; Sun et al., 2021; Xue et al., 2021). Thus, miR-21 as a potential diagnostic or therapeutic target for fibrosis diseases should be highlighted in future.

## Future Perspective of MICRORNAs in Hypertrophy of Ligamentum Flavum and Ossification of Ligamentum Flavum

### MicroRNA-Based Molecular Mechanisms

**Figure 2:**
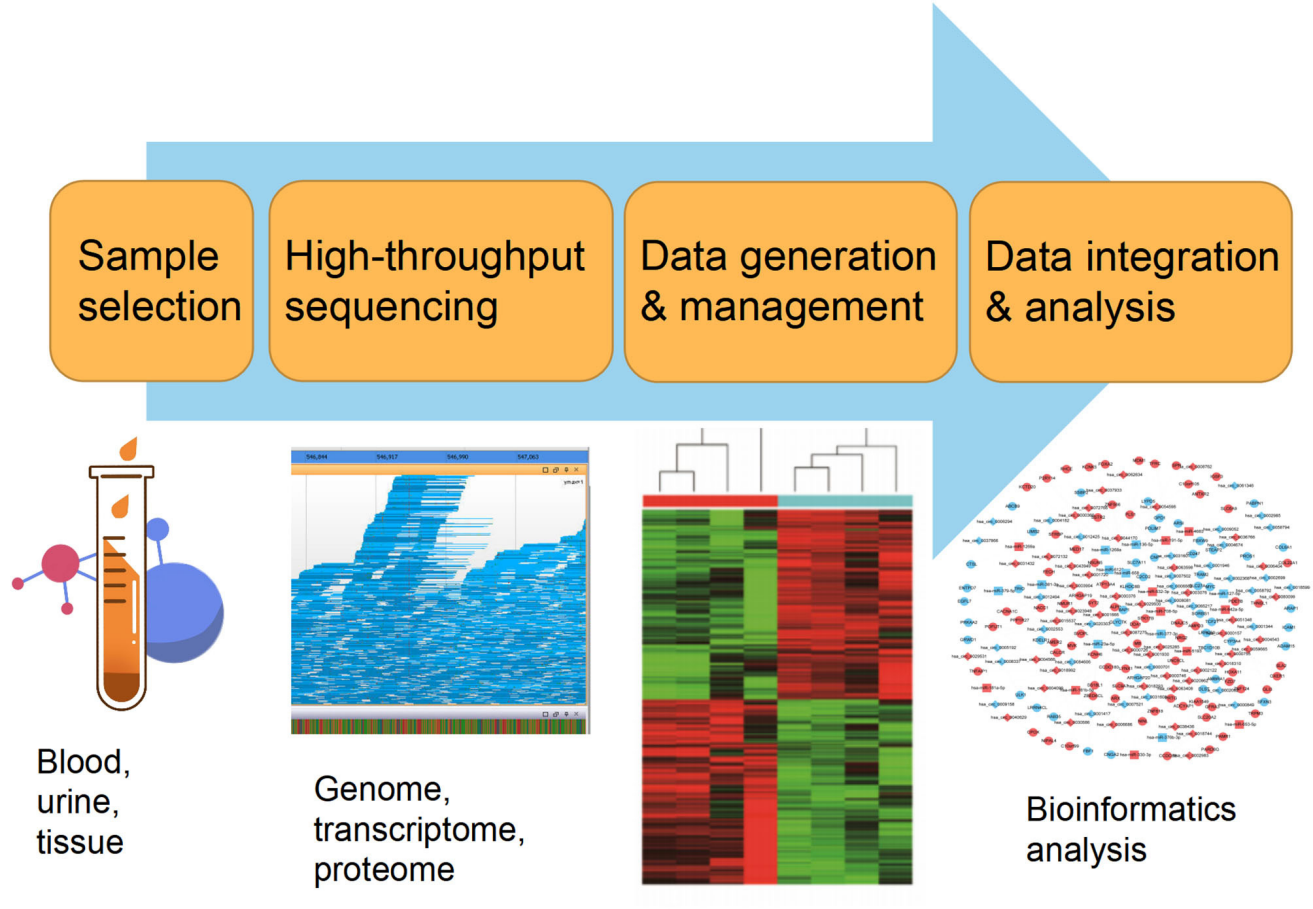
High-throughput biotechnological analysis workflow.

**Figure 3:**
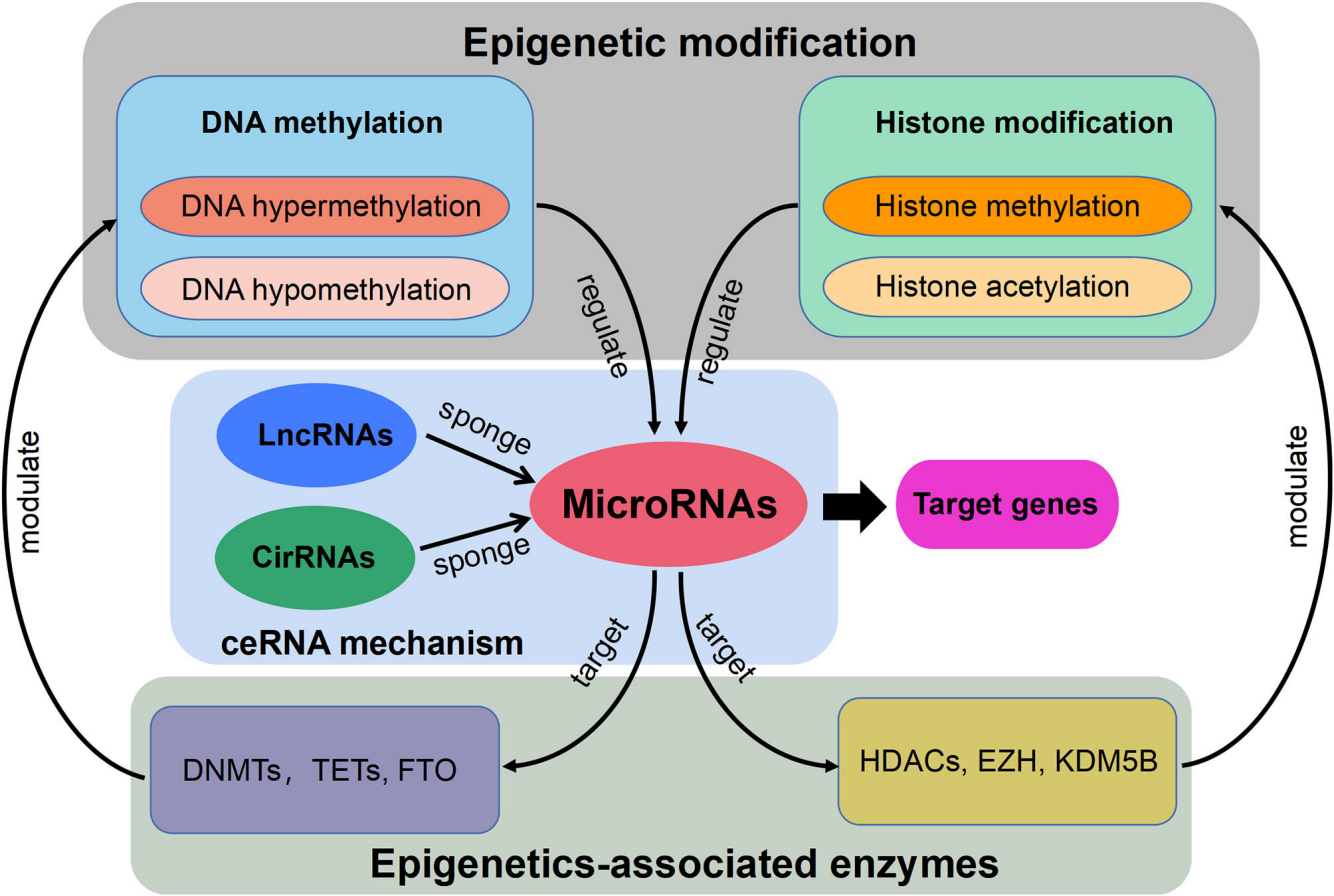
Schematic diagram of the miRNA-epigenetic feedback loop and the competitive endogenous RNA (ceRNA) mechanism.

Currently, the constant advancement of various high-throughput sequencing techniques will be particularly conducive to scientific researches for new molecular mechanisms of HLF/OLF (Guo et al., 2020) (Figure 2). ncRNAs include miRNA, lncRNAs, and circRNAs (Lekka and Hall, 2018). In most cases, miRNAs can interact with circRNAs or lncRNAs to regulate their functions. In turn, lncRNAs and circRNAs can act as a sponge to draw miRNAs, alleviating the inhibitory effect of miRNAs on their target genes, which is called the competitive endogenous RNA (ceRNA) mechanism (Yuan et al., 2021). The intricate regulatory networks and the widespread crosstalks among non-coding RNAs provide us with another direction to understand indepthly the molecular mechanism of HLF/OLF. Similarly, the lncRNA SNHG1/microRNA-320b/IFNGR1 network, lncRNA XIST/miR-17-5P/AHNAK/BMP2 signaling, and lncRNA MALAT1/miR-1/Connexin 43 network have been identified and validated in the development of OPLL (Liao et al., 2019; Yuan et al., 2019; Wang et al., 2020). However, sporadic sequencing and bioinformatics studies only focused on the individual ncRNA or predicted the ceRNA networks in the progression of HLF/OLF without further validation (Han et al., 2018; Kong et al., 2019; Wu et al., 2020). Besides ncRNAs, DNA methylation and histone modification are another two most widely studied forms of epigenetic regulations (Brookes and Shi, 2014; Berdasco and Esteller, 2019). miRNAs cannot only act as epigenetic modulators by targeting responsible epigenetic-associated enzymes, but the transcriptional regulation of miRNAs is also mediated by these epigenetic machinery, such as DNA methylation and histone modification (Gelato et al., 2016; Huang et al., 2019; Yao et al., 2019). Thus, the reciprocity interaction between miRNAs and other epigenetic regulatory forms can constitute the miRNA-epigenetic feedback loop (Figure 3), which has emerged as a novel mechanism of regulating multiple cellular processes, including osteogenesis differentiation (Li L. et al., 2018; Chen et al., 2019; Liu N. et al., 2020). Importantly, a previous study demonstrated that histone H3 modifications (e.g., H3K4me3, H3K9ac, and H3K18ac) might strongly link to the development of OLF (Hou et al., 2014). Therefore, exploring the link between miRNAs and histone modifications may improve our understanding of HLF/OLF pathogenesis. On the other hand, DNA methylation of miRNAs has been proven to hold particular regulation function in osteogenic differentiation (Allas et al., 2019; Yu et al., 2020). For example, Li et al. found that hypermethylation of miR-149 influenced the osteogenic differentiation of MSCs through SDF-1/CXCR4 signaling (Li et al., 2019). Interestingly, Fan et al. (Fan et al., 2020) performed a genome-wide DNA methylation analysis to establish an altered DNA methylation profiling in TOLF. Six differentially expressed methylated genes (HOXA10, SLC7A11, HOXA11AS, HOTAIR, TNIK, and IFITM1) were first identified, and further studies will explore the roles of these genes or interaction with miRNAs in the initiation and progression of TOLF (Fan et al., 2020). Additionally, possible new epigenetic mechanisms need to be explored, as well as the role of other classes of noncoding RNAs. Taken together, epigenetics, as an important bridge linking genetic and environmental factors, will be the focused area of in-depth research on the molecular mechanisms of HLF/OLF.

### MiRNA-Based Diagnostic Biomarkers

Generally, being integrated into microparticles (exosomes, microvesicles, and apoptotic bodies), or combined with specific proteins and lipoproteins make miRNAs highly stable and detectable in biological fluids (i.e., blood, serum, and plasma) (Hackl et al., 2016; Bottani et al., 2020). Furthermore, ample studies illustrated that a certain miRNA expression possessed tissue and pathology specificity in different kinds of diseases (Bartel, 2004). So far, circulating miRNAs have also been explored and identified as potential biomarkers for early non-invasive detection of several insidious congenital musculoskeletal disorders or age-associated bone diseases, such as lumbar degenerative disc disease (miR-155-5p) (Divi et al., 2020), OPLL (miR-10a, miR-210, and miR-563) (Xu et al., 2019), postmenopausal osteoporosis (miR-194-5p) (Ding et al., 2017), ankylosing spondylitis (miR-125a-5p, miR-151a-3p, miR-150-5p, and miR-451a) (Perez-Sanchez et al., 2018), adolescent idiopathic scoliosis (miR-122a-5p, miR-27a-5p, miR-223-5p, and miR-1306-3p) (García-Giménez et al., 2018), and osteoarthritis (miR-140-3p, miR-33b-3p, and miR-671-3p) (Ntoumou et al., 2017). Of course, numerous variables (e.g., sample types, manipulation, and detection techniques) in the pre-analytical, analytical, and post-analytical processes can inevitably affect miRNA quantification and validation. It is of great importance to formulate and follow detailed and standardized guidelines for extensive screenings and accurate identification of markedly differentially expressed miRNAs that are not related to other pathologies (Bottani et al., 2020). However, there are no preliminary studies on detecting and selecting circulating miRNAs as potential biomarkers for predisposition, diagnosis, or prognosis of hypertrophy and ossification of ligamentum flavum. Considering the insidious onset and progressive course of HLF/OLF, it will provide a research direction to investigate a single miRNA or a panel of miRNAs for evaluating the occurrence, recurrence, or progression of HLF/OLF in the future.

### MiRNA-Based Therapeutic Targets

To date, there is no particularly effective conservative treatment delaying or reversing the progression of HLF/OLF but surgical decompression. However, procedural risks and postoperative complications are very prominent (Hou et al., 2018; Liu Y. et al., 2020). MiRNAs are endogenous and multifunctional small molecules with numerous regulatory functions in various biological processes, and miRNA-based treatment methods present great potential for multiple diseases, such as cancer, infectious diseases, and autoimmune diseases. For example, the inhibition of specific miRNAs by modified oligonucleotide analogs may become a kind of promising therapy for HLF/OLF. Miravirsen, an anti-miR-122 oligonucleotide, has reached phase II clinical trials for chronic hepatitis C (CHC), and administration of various doses of miravirsen caused a substantial and sustained decline in plasma miR-122 levels without affecting the levels of other miRNAs in CHC patients (van der Ree et al., 2016). In addition, a variety of materials can be used as gene carriers for intracellular delivery of miRNAs (Gessler Dominic et al., 2019). For instance, liposome capsules, drug molecules with phospholipid bilayer vesicles, possess better biocompatibility and stability, which easily get into the cell through endocytosis and achieve gene delivery (Lane Rachel et al., 2017). Moreover, multiple nanosystems, such as organic/inorganic nanoparticles and polymer nanoparticles, have been introduced as functional nanocarriers to complete targeted and intelligent gene delivery by virtue of the physicochemical properties of different materials (Mao et al., 2019). With the rapid development of miRNA therapy in different fields, the preparation of new intelligent gene delivery systems will have broad prospects. Of course, multifarious concerns should be taken into account and addressed in the miRNA-based therapeutic research, such as the stability and the immunogenicity of miRNAs, delivery manners, proper dosing, target cell recognition, and intervening time, interval, and so on (Basak et al., 2016).

## Contemporary Challenges and Limitations of Microrna Researches

In this review, we systematically analyzed and summarized a wide range of supporting evidence on differentially expressed miRNAs and their roles in the process of ligamentum flavum degradation, which demonstrated a powerful relationship between miRNAs and the pathogenesis of OLF/HLF. Nevertheless, no definite miRNA has been identified as a clinically useful biomarker or as therapeutic target for OLF/HLF. The current research findings were restricted by a variety of uncontrollable factors. First, considering small population samples used for explorative studies, the biological differences between study samples may contribute to the discrepancies of miRNAs in plasma and tissue (van den Berg et al., 2017). Second, study designs fail to take the natural progression of hypertrophy and ossification of LF into consideration so as to influence expression difference of various miRNAs. Third, we found that miRNA expression might be tissue specific because miRNAs derived from HLF and OLF ligament tissue were significantly different on the whole. Moreover, diversified microarray platforms and sequencing techniques with inconsistent sensitivity and comparability were applied for the discovery phase, which might be responsible for the discrepancies between studies (Mestdagh et al., 2014; Dave et al., 2019). Finally, another concern is the lack of representative animal models of OLF/HLF used for extensive explorative and functional studies for screening miRNA profiles and selecting the most differentially expressed miRNAs. Accordingly, these existing findings need to be interpreted with caution.

## Conclusion

**Figure 4:**
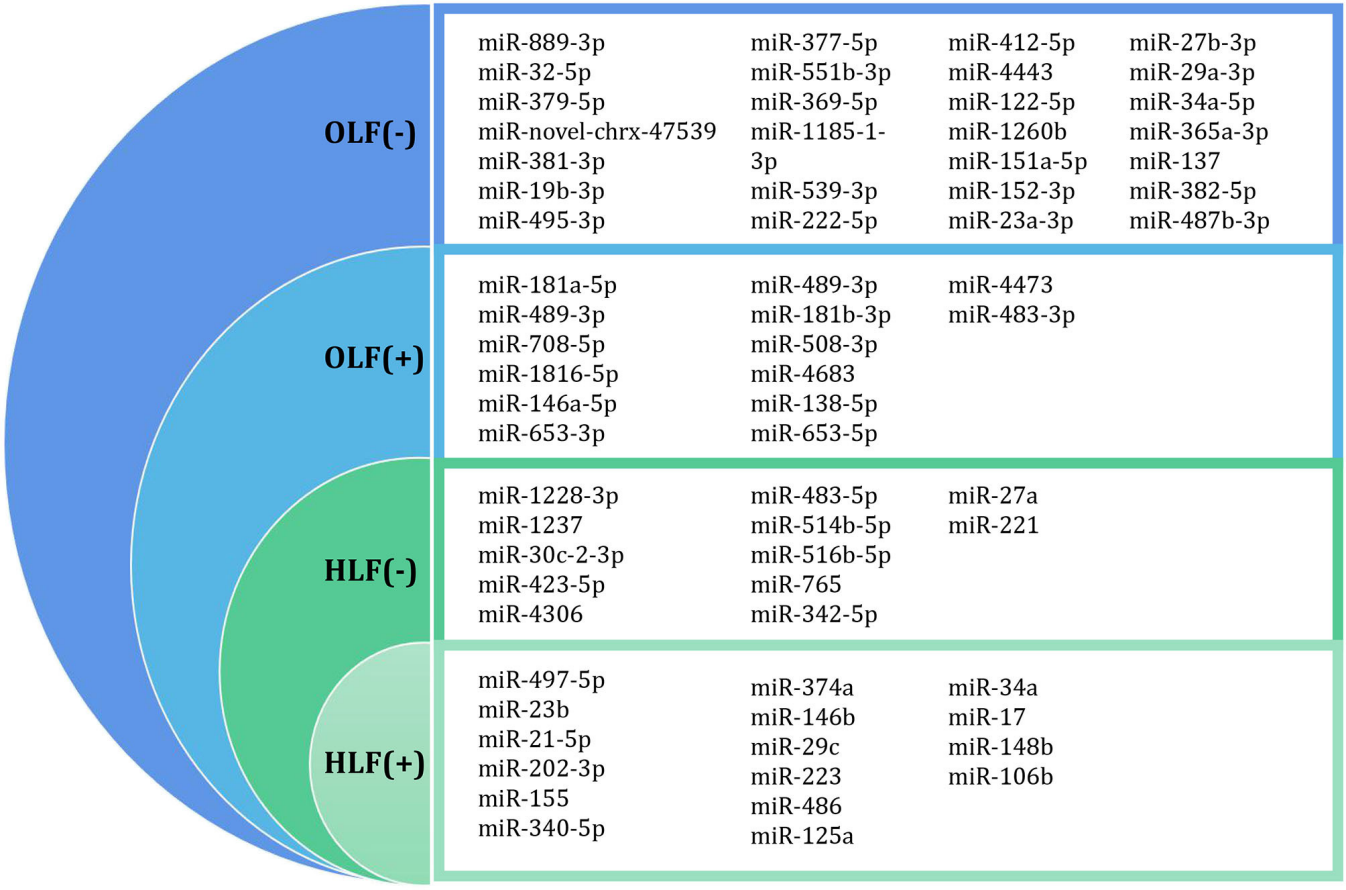
Differentially expressed miRNA profiling identified by microarray datasets or transcriptome sequencing between OLF/HLF samples vs. control samples. (+) represents upregulation of miRNAs, while (–) represents downregulation of miRNAs.

**Figure 5:**
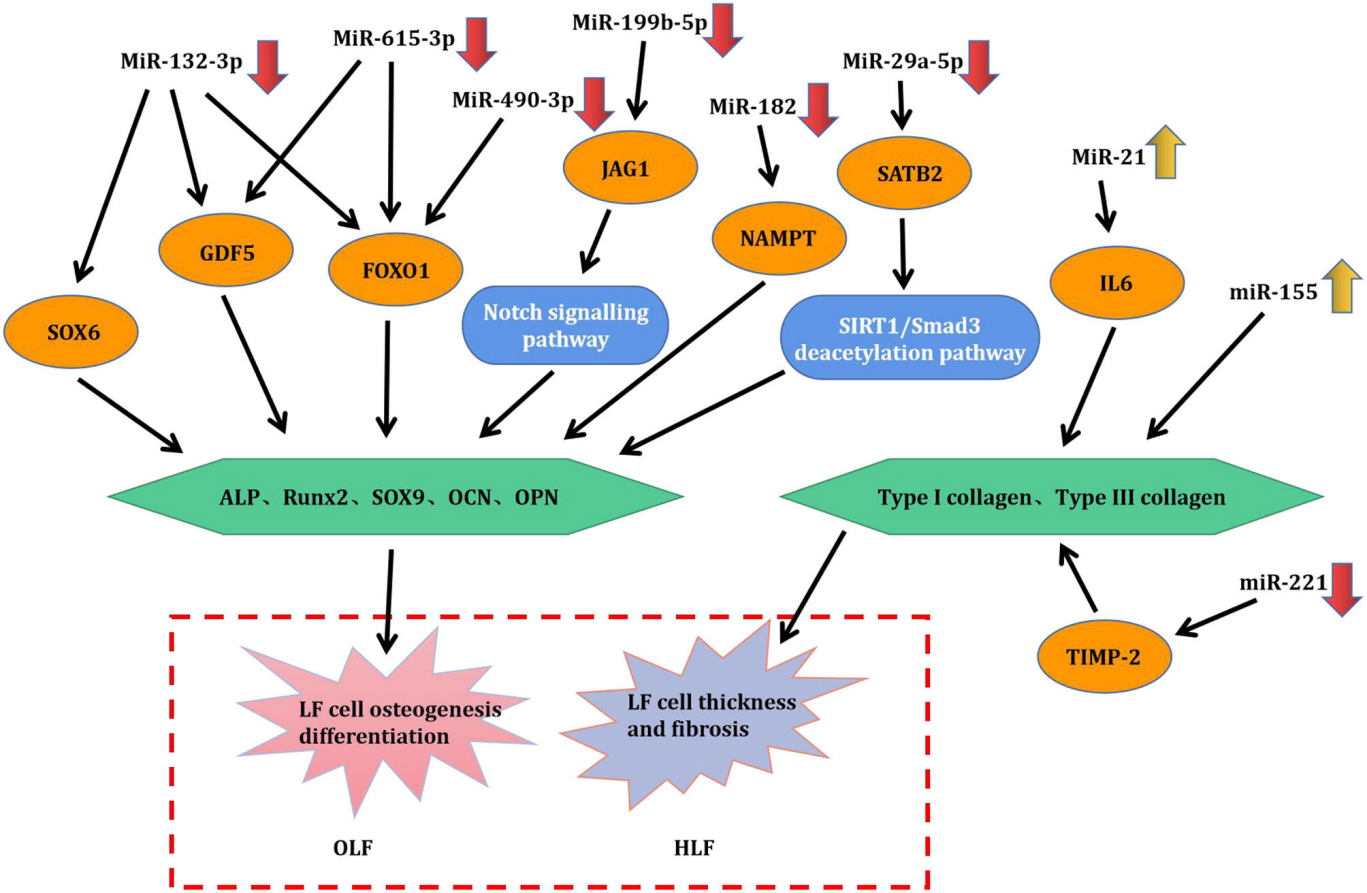
Pathogenic role and mechanism of deregulated miRNAs in HLF/OLF.

Through this review of miRNA-based publications, we summarized up-to-date evidence on the roles of miRNAs in the pathophysiology of hypertrophy and ossification of ligamentum flavum, though current investigations were just emerging. Explorative researches demonstrated that certain miRNAs in tissue were differentially expressed between patients with HLF/OLF and controls (Table 1 and Figure 4), while functional validations revealed that specific miRNAs played an important part by targeting certain downstream genes or via related molecular signaling pathways in the development of HLF/OLF (Table 2 and Figure 5). However, the direct role of these miRNAs that are abnormally expressed in the pathogenesis of HLF/OLF remains superficial and obscured. Even so, they might be conducive in uncovering the potential mechanisms of HLF/OLF and be promising to discover a group of practical biomarkers and develop a series of innovative therapies with more valuable translational research in the near future.

**Table 1:** Expression profiles of the miRNA in hypertrophy and ossification of ligamentum flavum.

**References**	**Country**	**Technique**	**Samples**		**DEM**	
			**Case group**	**Control group**	**Upregulated**	**Downregulated**
Han et al. (2018)	China	MicroRNA sequencing	4 OLF tissues	4 NLF tissues	12 miRNAs	16 miRNAs
Kong et al. (2019)	China	High throughput sequencing	4 OLF tissues	4 NLF tissues	25 miRNAs	56 miRNAs
Yayama et al. (2018)	Japan	MicroRNA sequencing	4 LF tissues of OPLL	4 LF tissues of CSM	NA	12 miRNAs
Wu et al. (2020)	China	MicroRNA sequencing	4 OLF tissues	4 NLF tissues	15 miRNAs	14 miRNAs
Xu Y. Q. et al. (2016)	China	MiRNA microarray	10 HLF tissues	10 LF tissues of LDH	15 miRNAs	3 miRNAs
Mori et al. (2017)	Japan	Agilent microarray	10 HLF tissues	10 LF tissues of LDH	1 miRNAs	9 miRNAs

**Table 2:** Functional characterization of specific miRNAs in hypertrophy and ossification of ligamentum flavum.

**References**	**Histopathology**	**MicroRNAs**	**Deregulation**	**Function**	**Targets/related pathways**
Qu et al. (2016b)	Thoracic OLF	miR-132-3p	Downregulated	Modulating osteogenic differentiation of LF cells	FOXO1, GDF5, SOX6
Qu et al. (2017)	Thoracic OLF	miR-199b-5p	Downregulated	Modulating osteogenic differentiation of LF cells	JAG1, Notch signaling pathway
Yang et al. (2018b)	Thoracic OLF	miR-490-3p	Downregulated	Modulating osteogenic differentiation of LF cells	FOXO1
Yin et al. (2017)	Lumbar OLF	miR-615-3p	Downregulated	Modulating osteogenic differentiation of LF cells	FOXO1, GDF5
Zhang et al. (2018)	Thoracic OLF	miR-182	Downregulated	Modulating osteogenic differentiation of LF cells	NAMPT
Feng et al. (2020)	Thoracic OLF	miR-29a-5p	Downregulated	Modulating osteogenic differentiation of LF cells	SATB2, SIRT1/Smad3 deacetylation pathway
Chen et al. (2014)	Lumbar HLF	miR-155	Upregulated	Modulating thickness and fibrosis process of LF cells	NA
Xu Y. Q. et al. (2016)	Lumbar HLF	miR-221	Downregulated	Modulating thickness and fibrosis process of LF cells	TIMP-2
Sun et al. (2017)	Lumbar HLF	miR-21	Upregulated	Inducing inflammation, modulating thickness and fibrosis process of LF cells	IL-6

## Data Availability Statement

The original contributions presented in the study are included in the article/supplementary material, further inquiries can be directed to the corresponding author/s.

## Author Contributions

ZC and BZ conceived and designed the study. BZ, GC, and XY collected the data. BZ, GC, XY, and TF analyzed and interpreted the patient data. BZ, GC, TF, and XC wrote and reviewed the paper. All authors read and approved the final manuscript.

## Conflict of Interest

The authors declare that the research was conducted in the absence of any commercial or financial relationships that could be construed as a potential conflict of interest.

## Abbreviations

LF: ligamentum flavum
HLF: hypertrophy of ligamentum flavum
OLF: ossification of ligamentum flavum
TSS: thoracic spinal stenosis
LSS: lumbar spinal stenosis
DM: diabetes mellitus
LDH: lumbar disc herniation
DEMs: differentially expressed miRNAs
OPLL: ossification of posterior longitudinal ligament
MeSH: Medical Subject Heading
PRISMA: the Preferred Reporting Items for Systematic Reviews and Meta-Analyses
ceRNA: competing endogenous RNA
NAMPT: nicotinamide phosphoribosyl transferase
SATB2: special AT-rich sequence-binding protein 2
TIMP: tissue inhibitors of matrix metalloproteinase
CHC: chronic hepatitis C.

## References

[B1] Allas L. Boumédiene K. Baugé C. (2019). Epigenetic dynamic during endochondral ossification and articular cartilage development. Bone 120, 523–532. 10.1016/j.bone.2018.10.004 30296494

[B2] Andersen G. B. Knudsen A. Hager H. Hansen L. L. Tost J. (2018). miRNA profiling identifies deregulated miRNAs associated with osteosarcoma development and time to metastasis in two large cohorts. Mol. Oncol. 12, 114–131. 10.1002/1878-0261.12154 29120535 PMC5748490

[B3] Artlett C. M. Sassi-Gaha S. Hope J. L. Feghali-Bostwick C. A. Katsikis P. D. (2017). Mir-155 is overexpressed in systemic sclerosis fibroblasts and is required for NLRP3 inflammasome-mediated collagen synthesis during fibrosis. Arthritis Res. Ther. 19:144. 10.1186/s13075-017-1331-z 28623945 PMC5473986

[B4] Bartel D. P. (2004). MicroRNAs: genomics, biogenesis, mechanism, and function. Cell 116, 281–297. 10.1016/S0092-8674(04)00045-5 14744438

[B5] Basak I. Patil K. S. Alves G. Larsen J. P. Møller S. G. (2016). microRNAs as neuroregulators, biomarkers and therapeutic agents in neurodegenerative diseases. Cell Mol. Life Sci. 73, 811–827. 10.1007/s00018-015-2093-x 26608596 PMC11108480

[B6] Berdasco M. Esteller M. (2019). Clinical epigenetics: seizing opportunities for translation. Nat. Rev. Genet. 20, 109–127. 10.1038/s41576-018-0074-2 30479381

[B7] Bicker S. Lackinger M. Weiß K. Schratt G. (2014). MicroRNA-132,−134, and−138: a microRNA troika rules in neuronal dendrites. Cell Mol. Life Sci. 71, 3987–4005. 10.1007/s00018-014-1671-7 25008044 PMC11113804

[B8] Bottani M. Banfi G. Lombardi G. (2020). The clinical potential of circulating miRNAs as biomarkers: present and future applications for diagnosis and prognosis of age-associated bone diseases. Biomolecules 10:E589. 10.3390/biom10040589 32290369 PMC7226497

[B9] Brookes E. Shi Y. (2014). Diverse epigenetic mechanisms of human disease. Annu. Rev. Genet. 48, 237–268. 10.1146/annurev-genet-120213-092518 25195505

[B10] Cavalli G. Heard E. (2019). Advances in epigenetics link genetics to the environment and disease. Nature 571, 489–499. 10.1038/s41586-019-1411-0 31341302

[B11] Chao Y. H. Huang S. Y. Yang R. C. Sun J. S. (2016). Tissue transglutaminase is involved in mechanical load-induced osteogenic differentiation of human ligamentum flavum cells. Connect Tissue Res. 57, 307–318. 10.1080/03008207.2016.1181062 27115725

[B12] Chaput C. D. Siddiqui M. Rahm M. D. (2019). Obesity and calcification of the ligaments of the spine: a comprehensive CT analysis of the entire spine in a random trauma population. Spine J. 19, 1346–1353. 10.1016/j.spinee.2019.03.003 30902702

[B13] Chen J. He G. Wang Y. Cai D. (2019). MicroRNA-223 promotes osteoblast differentiation of MC3T3-E1 cells by targeting histone deacetylase 2. Int. J. Mol. Med. 43, 1513–1521. 10.3892/ijmm.2018.4042 30628667

[B14] Chen J. Liu Z. Zhong G. Qian L. Li Z. Qiao Z.. (2014). Hypertrophy of ligamentum flavum in lumbar spine stenosis is associated with increased miR-155 level. Dis. Markers 2014:786543. 10.1155/2014/786543 24963214 PMC4052175

[B15] Coutinho de A. R. Ramos Y. F. M. Mahfouz A. den Hollander W. Lakenberg N. Houtman E.. (2019). RNA sequencing data integration reveals an miRNA interactome of osteoarthritis cartilage. Ann. Rheum. Dis. 78, 270–277. 10.1136/annrheumdis-2018-213882 30504444 PMC6352405

[B16] Dario A. B. Ferreira M. L. Refshauge K. M. Lima T. S. Ordoñana J. R. Ferreira P. H. (2015). The relationship between obesity, low back pain, and lumbar disc degeneration when genetics and the environment are considered: a systematic review of twin studies. Spine J. 15, 1106–1117. 10.1016/j.spinee.2015.02.001 25661432

[B17] Dave V. P. Ngo T. A. Pernestig A. K. Tilevik D. Kant K. Nguyen T.. (2019). MicroRNA amplification and detection technologies: opportunities and challenges for point of care diagnostics. Lab. Invest. 99, 452–469. 10.1038/s41374-018-0143-3 30542067

[B18] Ding H. Meng J. Zhang W. Li Z. Li W. Zhang M.. (2017). Medical examination powers miR-194-5p as a biomarker for postmenopausal osteoporosis. Sci. Rep. 7:16726. 10.1038/s41598-017-17075-w 29196685 PMC5711921

[B19] Divi S. N. Markova D. Z. Fang T. Guzek R. Kurd M. F. Rihn J. A.. (2020). Circulating miR-155-5p as a novel biomarker of lumbar degenerative disc disease. Spine 45, E499–E507. 10.1097/BRS.0000000000003322 31770330

[B20] Fan T. Meng X. Sun C. Yang X. Chen G. Li W.. (2020). Genome-wide DNA methylation profile analysis in thoracic ossification of the ligamentum flavum. J. Cell Mol. Med. 24, 8753–8762. 10.1111/jcmm.15509 32583558 PMC7412700

[B21] Feng F. Qiu H. Zhu D. Li X. Ning H. Yang D. (2020). miR-29a-5p targets SATB2 and regulates the SIRT1/Smad3 deacetylation pathway to inhibit thoracic ligamentum flavum cell osteogenesis. Spine 45, E1057–E1065. 10.1097/BRS.0000000000003505 32205703

[B22] Gao Y. Patil S. Qian A. (2020). The role of microRNAs in bone metabolism and disease. Int. J. Mol. Sci. 21:6081. 10.3390/ijms21176081 32846921 PMC7503277

[B23] García-Giménez J. L. Rubio-Belmar P. A. Peiró-Chova L. Hervás D. González-Rodríguez D. Ibañez-Cabellos J. S.. (2018). Circulating miRNAs as diagnostic biomarkers for adolescent idiopathic scoliosis. Sci. Rep. 8:2646. 10.1038/s41598-018-21146-x 29422531 PMC5805715

[B24] Gelato K. A. Shaikhibrahim Z. Ocker M. Haendler B. (2016). Targeting epigenetic regulators for cancer therapy: modulation of bromodomain proteins, methyltransferases, demethylases, and microRNAs. Exp. Opin. Ther. Targets 20, 783–799. 10.1517/14728222.2016.1134490 26799480

[B25] Gessler Dominic J. Tai Phillip W. L. Li J. Gao G. (2019). Intravenous infusion of AAV for widespread gene delivery to the nervous system. Methods Mol. Biol. 1950, 143–163. 10.1007/978-1-4939-9139-6_8 30783972 PMC7339923

[B26] Giulioni M. Zucchelli M. Damiani S. (2007). Thoracic myelopathy caused by calcified ligamentum flavum. Joint Bone Spine 74, 504–505. 10.1016/j.jbspin.2007.01.029 17709270

[B27] Gong K. Qu B. Liao D. Liu D. Wang C. Zhou J.. (2016). miR-132 regulates osteogenic differentiation via downregulating Sirtuin1 in a peroxisome proliferator-activated receptor β/δ-dependent manner. Biochem. Biophys. Res. Commun. 478, 260–267. 10.1016/j.bbrc.2016.07.057 27422605

[B28] Gu H. Wu L. Chen H. Huang Z. Xu J. Zhou K.. (2019). Identification of differentially expressed microRNAs in the bone marrow of osteoporosis patients. Am. J. Transl. Res. 11, 2940–2954. 31217865 PMC6556634

[B29] Guan H. Shang G. Cui Y. Liu J. Sun X. Cao W.. (2019). Long noncoding RNA APTR contributes to osteosarcoma progression through repression of miR-132-3p and upregulation of yes-associated protein 1. J. Cell Physiol. 234, 8998–9007. 10.1002/jcp.27572 30317613

[B30] Gulyaeva L. F. Kushlinskiy N. E. (2016). Regulatory mechanisms of microRNA expression. J. Transl. Med. 14:143. 10.1186/s12967-016-0893-x 27197967 PMC4873990

[B31] Guo H. Y. Guo M. K. Wan Z. Y. Song F. Wang H. Q. (2020). Emerging evidence on noncoding-RNA regulatory machinery in intervertebral disc degeneration: a narrative review. Arthritis Res. Ther. 22:270. 10.1186/s13075-020-02353-2 33198793 PMC7667735

[B32] Hackl M. Heilmeier U. Weilner S. Grillari J. (2016). Circulating microRNAs as novel biomarkers for bone diseases - Complex signatures for multifactorial diseases? Mol. Cell Endocrinol. 432, 83–95. 10.1016/j.mce.2015.10.015 26525415

[B33] Han Y. Hong Y. Li L. Li T. Zhang Z. Wang J.. (2018). A transcriptome-level study identifies changing expression profiles for ossification of the ligamentum flavum of the spine. Mol. Ther. Nucleic Acids 12, 872–883. 10.1016/j.omtn.2018.07.018 30161026 PMC6120750

[B34] Hayashi K. Suzuki A. Abdullah A. S. Terai H. Yamad K. Hoshino M.. (2017). Mechanical stress induces elastic fibre disruption and cartilage matrix increase in ligamentum flavum. Sci. Rep. 7:13092. 10.1038/s41598-017-13360-w 29026131 PMC5638934

[B35] Hou X. Chen Z. Sun C. Zhang G. Wu S. Liu Z. (2018). A systematic review of complications in thoracic spine surgery for ossification of ligamentum flavum. Spinal Cord 56, 301–307. 10.1038/s41393-017-0040-4 29284792

[B36] Hou X. Fan D. Sun C. Chen Z. (2014). Recombinant human bone morphogenetic protein-2-induced ossification of the ligamentum flavum in rats and the associated global modification of histone H3. J. Neurosurg. Spine 21, 334–341. 10.3171/2014.4.SPINE13319 24949905

[B37] Hu Z. Wang Y. Sun Z. Wang H. Zhou H. Zhang L.. (2015). miRNA-132-3p inhibits osteoblast differentiation by targeting Ep300 in simulated microgravity. Sci. Rep. 5:18655. 10.1038/srep18655 26686902 PMC4685444

[B38] Huang D. Cui L. Ahmed S. Zainab F. Wu Q. Wang X.. (2019). An overview of epigenetic agents and natural nutrition products targeting DNA methyltransferase, histone deacetylases and microRNAs. Food Chem. Toxicol. 123, 574–594. 10.1016/j.fct.2018.10.052 30408543

[B39] Inoue K. Deng Z. Chen Y. Giannopoulou E. Xu R. Gong S.. (2018). Bone protection by inhibition of microRNA-182. Nat. Commun. 9:4108. 10.1038/s41467-018-06446-0 30291236 PMC6173760

[B40] Jezek J. Sepitka J. Daniel M. Kujal P. Blankova A. Waldauf P.. (2020). The role of vascularization on changes in ligamentum flavum mechanical properties and development of hypertrophy in patients with lumbar spinal stenosis. Spine J. 20, 1125–1133. 10.1016/j.spinee.2020.03.002 32179155

[B41] Ji M. L. Jiang H. Zhang X. J. Shi P. L. Li C. Wu H.. (2018). Preclinical development of a microRNA-based therapy for intervertebral disc degeneration. Nat. Commun. 9:5051. 10.1038/s41467-018-07360-1 30487517 PMC6262020

[B42] Jiang X. Tsitsiou E. Herrick S. E. Lindsay M. A. (2010). MicroRNAs and the regulation of fibrosis. FEBS J. 277, 2015–2021. 10.1111/j.1742-4658.2010.07632.x 20412055 PMC2963651

[B43] Jung H. J. Suh Y. (2014). Circulating miRNAs in ageing and ageing-related diseases. J. Genet Genom. 41, 465–472. 10.1016/j.jgg.2014.07.003 25269672 PMC4354804

[B44] Kim K. M. Park S. J. Jung S. H. Kim E. J. Jogeswar G. Ajita J.. (2012). miR-182 is a negative regulator of osteoblast proliferation, differentiation, and skeletogenesis through targeting FoxO1. J. Bone Miner Res. 27, 1669–1679. 10.1002/jbmr.1604 22431396

[B45] Kim S. Ha K. Lee J. Kim Y. (2018). Prevalence and related clinical factors of thoracic ossification of the ligamentum flavum-a computed tomography-based cross-sectional study. Spine J. 18, 551–557. 10.1016/j.spinee.2017.08.240 28823939

[B46] Kloosterman W. P. Plasterk R. H. A. (2006). The diverse functions of microRNAs in animal development and disease. Dev. Cell 11, 441–450. 10.1016/j.devcel.2006.09.009 17011485

[B47] Kong D. Zhao Q. Liu W. Wang F. (2019). Identification of crucial miRNAs and lncRNAs for ossification of ligamentum flavum. Mol. Med. Rep. 20, 1683–1699. 10.3892/mmr.2019.10377 31257472 PMC6625436

[B48] Kong W. Yang H. He L. Zhao J. J. Coppola D. Dalton W. S.. (2008). MicroRNA-155 is regulated by the transforming growth factor beta/Smad pathway and contributes to epithelial cell plasticity by targeting RhoA. Mol. Cell Biol. 28, 6773–6784. 10.1128/MCB.00941-08 18794355 PMC2573297

[B49] Ladd-Acosta C. Fallin M. D. (2016). The role of epigenetics in genetic and environmental epidemiology. Epigenomics 8, 271–283. 10.2217/epi.15.102 26505319

[B50] Lane Rachel S. Haller F. Michael Chavaroche Anais A. E. Almond A. DeAngelis Paul L. (2017). Heparosan-coated liposomes for drug delivery. Glycobiology 27, 1062–1074. 10.1093/glycob/cwx070 29044377 PMC5881748

[B51] Lekka E. Hall J. (2018). Noncoding RNAs in disease. FEBS Lett. 592, 2884–2900. 10.1002/1873-3468.13182 29972883 PMC6174949

[B52] Li G. An J. Han X. Zhang X. Wang W. Wang S. (2019). Hypermethylation of microRNA-149 activates SDF-1/CXCR4 to promote osteogenic differentiation of mesenchymal stem cells. J. Cell Physiol. 234, 23485–23494. 10.1002/jcp.28917 31206187

[B53] Li G. Liu K. Du X. (2018). Long non-coding RNA TUG1 promotes proliferation and inhibits apoptosis of osteosarcoma cells by sponging miR-132-3p and upregulating SOX4 expression. Yonsei Med. J. 59, 226–235. 10.3349/ymj.2018.59.2.226 29436190 PMC5823824

[B54] Li L. Liu W. Wang H. Yang Q. Zhang L. Jin F.. (2018). Mutual inhibition between HDAC9 and miR-17 regulates osteogenesis of human periodontal ligament stem cells in inflammatory conditions. Cell Death Dis. 9:480. 10.1038/s41419-018-0480-6 29691366 PMC5915523

[B55] Liao X. Tang D. Yang H. Chen Y. Chen D. Jia L.. (2019). Long Non-coding RNA XIST may influence cervical ossification of the posterior longitudinal ligament through regulation of miR-17-5P/AHNAK/BMP2 signaling pathway. Calcif Tissue Int. 105, 670–680. 10.1007/s00223-019-00608-y 31511959

[B56] Liu N. Zhang Z. Li L. Shen X. Sun B. Wang R.. (2020). MicroRNA-181 regulates the development of ossification of posterior longitudinal ligament via epigenetic modulation by targeting PBX1. Theranostics 10, 7492–7509. 10.7150/thno.44309 32685001 PMC7359103

[B57] Liu Y. Qi Y. Diaty D. M. Zheng G. Shen X. Lin S.. (2020). Treatment for lumbar spinal stenosis secondary to ligamentum flavum hypertrophy using percutaneous endoscopy through interlaminar approach: a retrospective study. J. Orthop. Surg. Res. 15:337. 10.1186/s13018-020-01874-5 32811508 PMC7437061

[B58] Löhr M. Hampl J. A. Lee J. Y. Ernestus R. I. Deckert M. Stenzel W. (2011). Hypertrophy of the lumbar ligamentum flavum is associated with inflammation-related TGF-β expression. Acta Neurochir. 153, 134–141. 10.1007/s00701-010-0839-7 20960015

[B59] Makeyev E. V. Maniatis T. (2008). Multilevel regulation of gene expression by microRNAs. Science 319, 1789–1790. 10.1126/science.1152326 18369137 PMC3139454

[B60] Mao L. Gao M. Xue X. Yao L. Wen W. Zhang X.. (2019). Organic-inorganic nanoparticles molecularly imprinted photoelectrochemical sensor for α-solanine based on p-type polymer dots and n-CdS heterojunction. Anal. Chim. Acta. 1059, 94–102. 10.1016/j.aca.2019.01.039 30876637

[B61] Mestdagh P. Hartmann N. Baeriswyl L. Andreasen D. Bernard N. Chen C.. (2014). Evaluation of quantitative miRNA expression platforms in the microRNA quality control (miRQC) study. Nat. Methods 11, 809–815. 10.1038/nmeth.3014 24973947

[B62] Moon B. J. Kuh S. U. Kim S. Kim K. S. Cho Y. E. Chin D. K. (2015). Prevalence, distribution, and significance of incidental thoracic ossification of the ligamentum flavum in Korean patients with back or leg pain: MR-based cross sectional study. J. Korean Neurosurg. Soc. 58, 112–118. 10.3340/jkns.2015.58.2.112 26361526 PMC4564742

[B63] Mori T. Sakai Y. Kayano M. Matsuda A. Oboki K. Matsumoto K.. (2017). MicroRNA transcriptome analysis on hypertrophy of ligamentum flavum in patients with lumbar spinal stenosis. Spine Surg. Relat. Res. 1, 211–217. 10.22603/ssrr.1.2017-0023 31440636 PMC6698563

[B64] Morinaga J. Kadomatsu T. Miyata K. Endo M. Terada K. Tian Z.. (2016). Angiopoietin-like protein 2 increases renal fibrosis by accelerating transforming growth factor-β signaling in chronic kidney disease. Kidney Int. 89, 327–341. 10.1016/j.kint.2015.12.021 26806834

[B65] Nakamura T. Okada T. Endo M. Nakamura T. Oike Y. Mizuta H. (2015). Angiopoietin-like protein 2 promotes inflammatory conditions in the ligamentum flavum in the pathogenesis of lumbar spinal canal stenosis by activating interleukin-6 expression. Eur Spine J. 24, 2001–2009. 10.1007/s00586-015-3835-z 25735609

[B66] Ntoumou E. Tzetis M. Braoudaki M. Lambrou G. Poulou M. Malizos K.. (2017). Serum microRNA array analysis identifies miR-140-3p, miR-33b-3p and miR-671-3p as potential osteoarthritis biomarkers involved in metabolic processes. Clin. Epigenetics 9:127. 10.1186/s13148-017-0428-1 29255496 PMC5728069

[B67] Perez-Sanchez C. Font-Ugalde P. Ruiz-Limon P. Lopez-Pedrera C. Castro-Villegas M. C. Abalos-Aguilera M. C.. (2018). Circulating microRNAs as potential biomarkers of disease activity and structural damage in ankylosing spondylitis patients. Hum. Mol. Genet. 27, 875–890. 10.1093/hmg/ddy008 29329380

[B68] Pi C. Li Y. P. Zhou X. Gao B. (2015). The expression and function of microRNAs in bone homeostasis. Front Biosci. 20, 119–138. 10.2741/4301 25553443

[B69] Qu X. Chen Z. Fan D. Sun C. Zeng Y. (2016b). MiR-132-3p regulates the osteogenic differentiation of thoracic ligamentum flavum cells by inhibiting multiple osteogenesis-related genes. Int. J. Mol. Sci. 17:E1370. 10.3390/ijms17081370 27556448 PMC5000765

[B70] Qu X. Chen Z. Fan D. Sun C. Zeng Y. Guo Z.. (2017). MiR-199b-5p inhibits osteogenic differentiation in ligamentum flavum cells by targeting JAG1 and modulating the Notch signalling pathway. J. Cell Mol. Med. 21, 1159–1170. 10.1111/jcmm.13047 27957826 PMC5431140

[B71] Qu X. Chen Z. Fan D. Sun C. Zeng Y. Hou X.. (2016a). Notch signaling pathways in human thoracic ossification of the ligamentum flavum. J. Orthop. Res. 34, 1481–1491. 10.1002/jor.23303 27208800

[B72] Safak A. A. Is M. Sevinc O. Barut C. Eryoruk N. Erdogmus B.. (2010). The thickness of the ligamentum flavum in relation to age and gender. Clin. Anat. 23, 79–83. 10.1002/ca.20883 19941359

[B73] Saito T. Hara M. Kumamaru H. Kobayakawa K. Yokota K. Kijima K.. (2017). Macrophage infiltration is a causative factor for ligamentum flavum hypertrophy through the activation of collagen production in fibroblasts. Dis. Markers 30, 171–179. 10.1016/j.ajpath.2017.08.020 28935572

[B74] Shemesh S. Sidon E. Kaisler E. Sheinis D. Velkes S. Ohana N.. (2018). Diabetes mellitus is associated with increased elastin fiber loss in ligamentum flavum of patients with lumbar spinal canal stenosis: results of a pilot histological study. Eur. Spine J. 27, 1614–1622. 10.1007/s00586-017-5315-0 28980077

[B75] Shunzhi Y. Zhonghai L. Ning Y. (2017). Mechanical stress affects the osteogenic differentiation of human ligamentum flavum cells via the BMP-Smad1 signaling pathway. Mol. Med. Rep. 16, 7692–7698. 10.3892/mmr.2017.7543 28944874

[B76] Sidon E. Shemesh S. S. Mor-Yossef M. L. Wiesenfeld Y. Ohana N. Benayahu D. (2019). Molecular profile of ultrastructure changes of the ligamentum flavum related to lumbar spinal canal stenosis. J. Cell Biochem. 120, 11716–11725 10.1002/jcb.28451 30825230

[B77] Silahtaroglu A. Stenvang J. (2010). MicroRNAs, epigenetics and disease. Essays Biochem. 48, 165–185. 10.1042/bse0480165 20822493

[B78] Skinner M. K. Manikkam M. Guerrero-Bosagna C. (2010). Epigenetic transgenerational actions of environmental factors in disease etiology. Trends Endocrinol. Metab. 21, 214–222. 10.1016/j.tem.2009.12.007 20074974 PMC2848884

[B79] Sugimoto K. Nakamura T. Tokunaga T. Uehara Y. Okada T. Taniwaki T.. (2018). Matrix metalloproteinase promotes elastic fiber degradation in ligamentum flavum degeneration. PLoS ONE 13:e0200872. 10.1371/journal.pone.0200872 30067795 PMC6070248

[B80] Sun C. Tian J. Liu X. Guan G. (2017). MiR-21 promotes fibrosis and hypertrophy of ligamentum flavum in lumbar spinal canal stenosis by activating IL-6 expression. Biochem. Biophys. Res. Commun. 490, 1106–1111. 10.1016/j.bbrc.2017.06.182 28669725

[B81] Sun C. Wang Z. Tian J. W. Wang Y. H. (2018). Leptin-induced inflammation by activating IL-6 expression contributes to the fibrosis and hypertrophy of ligamentum flavum in lumbar spinal canal stenosis. Biosci. Rep. 38:BSR20171214. 10.1042/BSR20171214 29436483 PMC5874260

[B82] Sun C. Zhang H. Wang X. Liu X. (2020). Ligamentum flavum fibrosis and hypertrophy: molecular pathways, cellular mechanisms, and future directions. FASEB J. 34, 9854–9868. 10.1096/fj.202000635R 32608536

[B83] Sun J. Shi L. Xiao T. Xue J. Li J. Wang P.. (2021). microRNA-21, via the HIF-1α/VEGF signaling pathway, is involved in arsenite-induced hepatic fibrosis through aberrant cross-talk of hepatocytes and hepatic stellate cells. Chemosphere 266:129177. 10.1016/j.chemosphere.2020.129177 33310519

[B84] Tsay H. C. Yuan Q. Balakrishnan A. Kaiser M. Möbus S. Kozdrowska E.. (2019). Hepatocyte-specific suppression of microRNA-221-3p mitigates liver fibrosis. J. Hepatol. 70, 722–734. 10.1016/j.jhep.2018.12.016 30582979

[B85] van den Berg N. W. E. Kawasaki M. Berger W. R. Neefs J. Meulendijks E. Tijsen A. J.. (2017). MicroRNAs in atrial fibrillation: from expression signatures to functional implications. Cardiovasc. Drugs Ther. 31, 345–365. 10.1007/s10557-017-6736-z 28752208 PMC5550535

[B86] van der Ree M. H. van der Meer A. J. van Nuenen A. C. de Bruijne J. Ottosen S. Janssen H. L.. (2016). Miravirsen dosing in chronic hepatitis C patients results in decreased microRNA-122 levels without affecting other microRNAs in plasma. Aliment Pharmacol. Ther. 43, 102–113. 10.1111/apt.13432 26503793

[B87] Waki T. Lee S. Y. Niikura T. Iwakura T. Dogaki Y. Okumachi E.. (2015). Profiling microRNA expression in fracture nonunions: potential role of microRNAs in nonunion formation studied in a rat model. Bone Joint J. 97-B:1144–1151. 10.1302/0301-620X.97B8.34966 26224835

[B88] Wang G. Kwan B. C. Lai F. M. Chow K. M. Li P. K. Szeto C. C. (2011). Elevated levels of miR-146a and miR-155 in kidney biopsy and urine from patients with IgA nephropathy. Dis. Markers 30, 171–179. 10.1155/2011/304852 21694443 PMC3825242

[B89] Wang L. Wu F. Song Y. Li X. Wu Q. Duan Y.. (2016). Long noncoding RNA related to periodontitis interacts with miR-182 to upregulate osteogenic differentiation in periodontal mesenchymal stem cells of periodontitis patients. Cell Death Dis. 7:e2327. 10.1038/cddis.2016.125 27512949 PMC5108307

[B90] Wang Y. Niu H. Liu Y. Yang H. Zhang M. Wang L. (2020). Promoting effect of long non-coding RNA SNHG1 on osteogenic differentiation of fibroblastic cells from the posterior longitudinal ligament by the microRNA-320b/IFNGR1 network. Cell Cycle 19, 2836–2850. 10.1080/15384101.2020.1827188 33017569 PMC7714528

[B91] Wu W. Chen Y. Yang Z. Zhang F. Ru N. Wu B.. (2020). The role of gene expression changes in ceRNA network underlying ossification of ligamentum flavum development. DNA Cell Biol. 39, 1162–1171. 10.1089/dna.2020.5446 32559389

[B92] Xu C. Chen Y. Zhang H. Chen Y. Shen X. Shi C.. (2016). Integrated microRNA-mRNA analyses reveal OPLL specific microRNA regulatory network using high-throughput sequencing. Sci. Rep. 6:21580. 10.1038/srep21580 26868491 PMC4751494

[B93] Xu C. Zhang H. Zhou W. Wu H. Shen X. Chen Y.. (2019). MicroRNA-10a,−210, and−563 as circulating biomarkers for ossification of the posterior longitudinal ligament. Spine J. 19, 735–743. 10.1016/j.spinee.2018.10.008 30352301

[B94] Xu Y. Q. Zhang Z. H. Zheng Y. F. Feng S. Q. (2016). MicroRNA-221 regulates hypertrophy of ligamentum flavum in lumbar spinal stenosis by targeting TIMP-2. Spine 41, 275–282. 10.1097/BRS.0000000000001226 26571175

[B95] Xue J. Xiao T. Wei S. Sun J. Zou Z. Shi M.. (2021). miR-21-regulated M2 polarization of macrophage is involved in arsenicosis-induced hepatic fibrosis through the activation of hepatic stellate cells. J. Cell Physiol. 10.1002/jcp.30288. [Epub ahead of print]. 33481270

[B96] Yabe Y. Hagiwara Y. Ando A. Tsuchiya M. Minowa T. Takemura T. (2015). Chondrogenic and fibrotic process in the ligamentum flavum of patients with lumbar spinal canal stenosis. Spine 40, 429–435. 10.1097/BRS.0000000000000795 25627290

[B97] Yan L. Wang L. Z. Xiao R. Cao R. Pan B. Lv X. Y.. (2020). Inhibition of microRNA-21-5p reduces keloid fibroblast autophagy and migration by targeting PTEN after electron beam irradiation. Lab. Invest. 100, 387–399. 10.1038/s41374-019-0323-9 31558773

[B98] Yang X. Chen Z. Meng X. Sun C. Li M. Shu L.. (2018a). Angiopoietin-2 promotes osteogenic differentiation of thoracic ligamentum flavum cells via modulating the Notch signaling pathway. PLoS ONE 13:e0209300. 10.1371/journal.pone.0209300 30557327 PMC6296551

[B99] Yang X. Qu X. Meng X. Li M. Fan D. Fan T.. (2018b). MiR-490-3p inhibits osteogenic differentiation in thoracic ligamentum flavum cells by targeting FOXO1. Int. J. Biol. Sci. 14, 1457–1465. 10.7150/ijbs.26686 30262997 PMC6158729

[B100] Yao Q. Chen Y. Zhou X. (2019). The roles of microRNAs in epigenetic regulation. Curr. Opin. Chem. Biol. 51, 11–17. 10.1016/j.cbpa.2019.01.024 30825741

[B101] Yayama T. Mori K. Okumura N. Nishizawa K. Kumagai K. Nakamura A.. (2018). Wnt signaling pathway correlates with ossification of the spinal ligament: a microRNA array and immunohistochemical study. J. Orthop. Sci. 23, 26–31. 10.1016/j.jos.2017.09.024 29102319

[B102] Yayama T. Uchida K. Kobayashi S. Kokubo Y. Sato R. Nakajima H.. (2007). Thoracic ossification of the human ligamentum flavum: histopathological and immunohistochemical findings around the ossified lesion. J. Neurosurg. Spine 7, 184–193. 10.3171/SPI-07/08/184 17688058

[B103] Ye S. Kwon W. K. Bae T. Kim S. Lee J. B. Cho T. H.. (2019). CCN5 reduces ligamentum flavum hypertrophy by modulating the TGF-β pathway. J. Orthop. Res. 37, 2634–2644. 10.1002/jor.24425 31334871 PMC6899892

[B104] Yin J. Zhuang G. Zhu Y. Hu X. Zhao H. Zhang R.. (2017). MiR-615-3p inhibits the osteogenic differentiation of human lumbar ligamentum flavum cells via suppression of osteogenic regulators GDF5 and FOXO1. Cell Biol Int. 41, 779–786. 10.1002/cbin.10780 28460412

[B105] Yu L. Xia K. Cen X. Huang X. Sun W. Zhao Z.. (2020). DNA methylation of noncoding RNAs: new insights into osteogenesis and common bone diseases. Stem Cell Res. Ther. 11:109. 10.1186/s13287-020-01625-7 32143708 PMC7060611

[B106] Yuan X. Guo Y. Chen D. Luo Y. Chen D. Miao J.. (2019). Long non-coding RNA MALAT1 functions as miR-1 sponge to regulate Connexin 43-mediated ossification of the posterior longitudinal ligament. Bone 127, 305–314. 10.1016/j.bone.2019.06.019 31280017

[B107] Yuan X. Shi L. Chen Y. (2021). Non-coding RNAs in ossification of spinal ligament. Eur. Spine J. 10.1007/s00586-020-06687-y. [Epub ahead of print]. 33387048

[B108] Zhang C. Chen Z. Meng X. Li M. Zhang L. Huang A. (2017). The involvement and possiblemechanism of pro-inflammatory tumor necrosis factor alpha (TNF-α) in thoracic ossification of the ligamentum flavum. PLoS ONE 12:e0178986. 10.1371/journal.pone.0178986 28575129 PMC5456390

[B109] Zhang K. Sun W. Liu X. Zhao C. Li H. Sun X.. (2017). Hypertrophy and fibrosis of the ligamentum flavum in lumbar spinal stenosis is associated with increased expression of LPA and LPAR1. Clin Spine Surg. 30, E189–E191. 10.1097/BSD.0000000000000048 28323698

[B110] Zhang Q. Shen Y. Jiang Y. Zhao S. Zhou D. Xu N. (2018). Overexpression of miR-182 inhibits ossification of ligamentum flavum cells by targeting NAMPT. Exp Cell Res. 367, 119–131. 10.1016/j.yexcr.2018.03.008 29601800

[B111] Zhao R. Li Y. Lin Z. Wan J. Xu C. Zeng Y.. (2016). miR-199b-5p modulates BMSC osteogenesis via suppressing GSK-3β/β-catenin signaling pathway. Biochem. Biophys. Res. Commun. 477, 749–754. 10.1016/j.bbrc.2016.06.130 27363340

[B112] Zhou Y. Ng D. Richards A. M. Wang P. (2020). microRNA-221 inhibits latent TGF-β1 activation through targeting thrombospondin-1 to attenuate kidney failure-induced cardiac fibrosis. Mol. Ther. Nucleic Acids 22, 803–814. 10.1016/j.omtn.2020.09.041 33230477 PMC7645417

